# Controllable Friction on Graphene via Adjustable Interfacial Contact Quality

**DOI:** 10.1002/advs.202303013

**Published:** 2023-09-03

**Authors:** Wen Wang, Yu Zhang, Zhihong Li, Linmao Qian

**Affiliations:** ^1^ School of Mechanical Engineering Southwest Jiaotong University Chengdu 610031 China; ^2^ Key Laboratory of Microelectronic Devices and Circuits (MOE) Institute of Microelectronics Peking University Beijing 100871 China

**Keywords:** contact quality, frictional storage medium, grapehene, out‐of‐plane tapping

## Abstract

Despite the numerous unique properties revealed through tribology research on graphene, the development of applications that utilize its rich tribological properties remains a long‐sought goal. In this article, a novel approach for reversible patterning of graphene's frictional properties using out‐of‐plane mechanical tapping is presented. The friction force between the atomic force microscopy (AFM) tip and the graphene film is increased by up to a factor of two, which can be attributed to variations in the interfacial binding strength between the graphene and substrate through the tapping process. The reversible and repeatable frictional properties of graphene make it a promising material for information storage applications with a high storage capacity of ≈1600 GB inch^−2^, allowing for direct writing and erasing of information, akin to a blackboard. These findings highlight the potential for friction tuning in lamellar materials and emphasize the significance of understanding nanoscale friction on graphene surfaces.

## Graphical abstract

The reversible and repeatable frictional properties of graphene induced by out‐of‐plane mechanical tapping make it a promising material for information storage applications with a high storage capacity of ≈1600 GB inch^−2^, allowing for direct writing and erasing of information, akin to a blackboard.

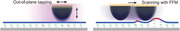

## 1 Introduction

Atomically thin 2D lamellar materials, including graphene, hexagonal boron nitride (h‐BN), transition metal dichalcogenides (TMD), and MAXene, possess unique physical and mechanical properties that have the potential to advance a wide range of nanotechnology applications.^[^ ^1^, ^2^, ^3^, ^4^ ^]^ Graphene, in particular, shows promise in nonvolatile memory devices,^[^ ^5^, ^6^ ^]^ light‐emitting diodes,^[^ ^7^ ^]^ and field‐effect transistors.^[^ ^8^ ^]^ Moreover, graphene and its derivatives are widely used as solid lubricants and protective films to improve the reliability and lifespan of sliding components.^[^ ^4^, ^9^, ^10^ ^]^

Previous studies in the field of tribology in the past years have primarily focused on the graphene's frictional properties on the basal plane and across step edges, which have revealed numerous interesting phenomena such as the dependence of friction on factors like thickness, crystalline orientation, sliding direction, and binding strength to the substrate, etc.^[^ ^11^, ^12^, ^13^, ^14^, ^15^, ^16^, ^17^, ^18^, ^19^, ^20^, ^21^, ^22^ ^]^ Especially, the intrinsic weak interlayer van der Waals interaction and low out‐of‐plane bending rigidity of 2D materials result in a lifting of the top layer in front of the scanning tip, that is, the puckering effect,^[^ ^11^, ^15^, ^22^ ^]^ leading to friction increasing with decreasing sample thickness.^[^ ^11^, ^15^, ^23^ ^]^ Moreover, the contact quality between the top layer and the scanning tip evolves with scanning time, with friction force gradually increasing for a few initial atomic periods before reaching a constant value.^[^ ^20^, ^22^ ^]^ The contact quality and puckering effects become more pronounced as the deformation compliance of 2D materials increases and/or the binding between the 2D membranes and the substrate decreases.^[^ ^17^, ^20^, ^24^, ^25^ ^]^

Despite the mechanisms of friction on graphene based on interfacial contact quality has been well documented from a fundamental perspective, the modulation and utilization of contact quality to intentionally alter the frictional properties remain largely unexplored.^[^ ^26^, ^27^ ^]^ In this study, we propose a straightforward and efficient method to manipulate friction on graphene by applying out‐of‐plane mechanical tapping using an atomic force microscopy (AFM) tip. This tapping process effectively modifies the binding strength between the graphene and the substrate, as evidenced by discernible changes in the friction force signal. Impressively, our experimental results demonstrate that friction on graphene can be modulated by a factor of up to two. A crucial aspect of our approach is the complete reversibility of the changes in binding strength, enabling precise control over stored contact information, reminiscent of a blackboard. Significantly, this method holds substantial potential for information storage applications, offering a remarkable capacity of ≈1600 GB inch^−2^ for digital data storage by using a commercial available tip with the typical radius of 10 nm.

## 2 Experimental Section

**Figure 1:**
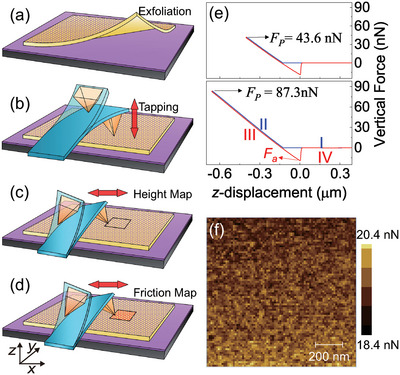
Sample fabrication, representative vertical force–distance curves and adhesion force map between AFM tip and graphene. a) Graphene films were prepared by mechanical exfoliation onto a clean SiO_2_/Si substrate. b) An Si_3_N_4_ AFM tip was employed to perform out‐of‐plane mechanical tapping on the graphene surface. The FFM with lateral force detection was used to analyze the c) topographical and d) tribological changes of the graphene after tapping. e) Representative vertical force‐distance curves during the tapping process with the pre‐defined tapping force of $F_{P}=43.6$ nN and $F_{P}=87.3$ nN. f) Adhesion force maps ($F_{a}$) with dimensions of 1μm×1μm reveals the spatial distribution of the adhesion forces between the AFM tip and graphene.

**Figure** **1**a–d depict the cartoon schematics of the sample fabrication process. First, graphene films were mechanically exfoliated onto a clean silicon substrate coated with a 300 nm thick layer of SiO_2_ (Figure 1a). To improve the efficiency of the preparation, the SiO_2_/Si substrate was pre‐treated with O_2_ plasma for 5 s, unless otherwise specified, prior to exfoliation. Subsequently, mechanical tapping was applied to the graphene surface in an out‐of‐plane (*z*) direction using a Si_3_N_4_ tip (MSCT from Bruker), as shown in Figure 1b. Finally, a contact mode friction force microscope (FFM) with lateral force detection was utilized to analyze the tribological and topographical changes of the graphene after tapping, as depicted in Figure 1c,d.

The AFM/FFM system (MFP‐3D, Oxford) was employed for all experiments, conducted in an ambient environment with a controlled relative humidity (RH) of 45% to 50% and a temperature of 23 ± 3 °C, unless otherwise specified. The lateral force values reported in this study were calibrated using the wedge calibration method.^[^ ^28^ ^]^ The zero applied normal load was defined as the force acting on the cantilever at its equilibrium deflection. Unless stated otherwise, the out‐of‐plane tapping and FFM scanning were performed at fixed rates of 8 and 1 Hz, respectively. Throughout the experiments, the observed topography and adhesion force signals remained remarkably stable, indicating the absence of significant wear.

It is important to note that the mechanical tapping process employed in the study shares similarities with the typical tapping mode in atomic force microscopy. In conventional tapping mode AFM, the cantilever oscillated at its resonant frequency, and the interaction force between the tip and the sample surface was deliberately kept very weak to prevent damage to the sample surface. However, in the mechanical out‐of‐plane tapping method, a slightly larger tapping force was applied in the range of several tenths of nanonewtons. This mechanical out‐of‐plane tapping process was achieved by continuously acquiring force–distance curves along the *z*‐direction, systematically acquiring point by point with a step size of typically 10 nm within a specific area. In this process, the AFM tip was repeatedly brought into contact with the graphene surface using a predefined tapping force ($F_{P}$), and then retracted, generating a series of force–distance curves. The mechanical tapping process closely resembles the adhesion force measurement process, with the main distinction being that in adhesion force measurement, the process was repeated at the same position, whereas in mechanical tapping, the measurement was repeated at subsequent positions.

Figure 1e displays typical force–distance curves obtained during the out‐of‐plane tapping process with predefined tapping forces of $F_{P}=43.6$ nN (top panel) and $F_{P}=87.3$ nN (bottom panel), along the *z* direction. In principle, the vertical force curve during tapping can be divided into four stages: I) Initially, the vertical force is zero until the AFM tip comes into contact with the sample surface. II) A nearly linear dependence of the vertical force on *z*‐displacement is observed until the predefined tapping force $F_{P}$ is reached, characterized by the predominant effects of cantilever bending. III) The vertical force shows a nearly linear dependence on displacement during retracting, before reaching the adhesion force $F_{a}$. IV) Finally, the vertical force returns to zero when the AFM tip is detached from the sample surface. In Figure 1f, typical $F_{a}$ force maps of 1μm×1μm are presented, indicating a uniform sample surface and a stable tapping process.

As observed in previous studies,^[^ ^29^, ^30^ ^]^ the exposure of graphene to ambient conditions for several days led to the adsorption of contaminants and the formation of quasi‐periodic ripples on the graphene surface. These adsorbates can influence the friction behavior of graphene and weaken the interaction between the AFM tip and graphene. To mitigate the potential influence of adsorbates, all measurements in this study were conducted on newly exfoliated graphene samples and completed within 12 h of exfoliation. Although the specific influence of adsorbates on friction patterning was not investigated in this study, it was observeed that frictional patterns on graphene sheets exposed to ambient conditions for several days were not as distinct as those on newly exfoliated samples. This observation suggests that the presence of adsorbates may reduce the tip‐sample interaction, resulting in less pronounced frictional patterns.

## 3 Results and Discussion

**Figure 2:**
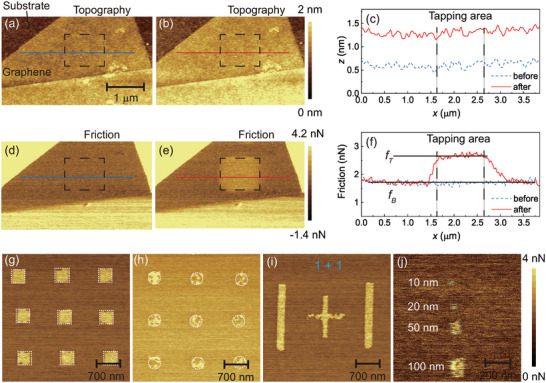
In situ patterning and characterization of the graphene film using out‐of‐plane tapping. a,b) Topography scans of the graphene surface obtained before and after tapping, which were obtained in contact mode operation at a normal force of 1 nN and a scan velocity of 10 μm s^−1^. The dashed rectangles indicate the tapping area. In both cases we find that the surfaces are atomically flat with no apparent differences between the two images. c) Cross‐section lines through the tapping area shown in (a) and (b) further demonstrate that the tapping process hardly change the surface topography. d,e) The simultaneously recorded friction signals before and after tapping. After tapping, the graphene surface exhibits a clear rectangle higher friction region. f) The cross‐section friction force lines through the tapping area shown in (d) and (e). We utilized the tapping approach to g) pattern rectangles with the size of 300 nm × 300 nm, h) circulars with the radius of 150 nm, and i) a mathematical expression “1+1”. j) The minimum achievable pattern size using our method was ≈10 nm.

To effectively showcase the novel findings, we present representative topographic and friction maps before and after out‐of‐plane tapping, acquired using contact mode FFM with a normal force of 1 nN and a scan velocity of 10 μm s^−1^. The images were taken over an area of 4 μm × 3 μm. Prior to tapping, the topography (**Figure** **2**a) and friction (Figure 2d) maps of the graphene on SiO_2_/Si substrate appeared relatively uniform, with no prominent features observed except for a few small glue clusters. Subsequently, a Si_3_N_4_ tip was employed to continuously tap on the graphene surface with a predefined tapping force of $F_{P}=$17.5 nN, as highlighted by the rectangles in Figure 2a–e. Following the tapping process, contact mode FFM was again employed to characterize the topography and friction properties. Notably, no significant changes in topography were observed (Figure 2b), while a distinct rectangular region exhibiting higher friction was evident (Figure 2e). Cross‐sectional profiles taken through the patterned area further confirmed the absence of significant topographical changes (Figure 2c), while clearly showing an elevated friction state within the tapping region (Figure 2f). For comparison, we conducted an additional experiment where we first performed scanning on the graphene using contact mode FFM and then scanned a larger area to verify the change of friction. Consistent with our findings, we observed no significant difference in friction properties, further supporting that the friction enhancement originates from the out‐of‐plane tapping process (see Figure S3, Supporting Information).

We further demonstrate the capabilities of our tapping method by successfully patterning the graphene film into various shapes, including rectangles with a size of 300 nm × 300 nm (Figure 2g), circles with a radius of 150 nm (Figure 2h), and even a representation of the mathematical expression “1+1” (Figure 2i). Notably, the minimum achievable pattern size using our method was ≈10 nm (Figure 2j), which is in close proximity to the size of the AFM tip radius. This suggests that the minimum size of the pattern may be influenced by the size of the AFM tip. It is important to emphasize that Figure 2g–j correspond to the friction maps obtained during the tapping process. Based on the minimum storage unit size of 10 nm× 10 nm, the storage density per square inch is estimated to be ≈1600 GB inch^−^2 (see Supporting Information for more information).

**Figure 3:**
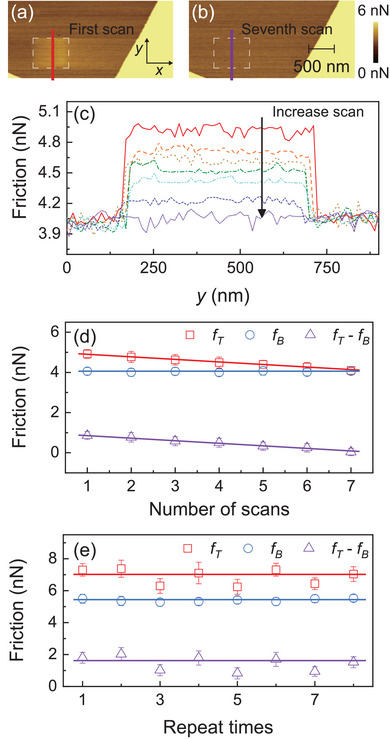
Data erasability and rewritability. a,b) The recorded friction force maps during the first and seventh scan with a normal force of 22 nN and scan velocity of 12 μm s^−1^. The pre‐written square in the friction map appear clearly on the first scan and finally disappears in the seventh scan. c) The typical cross‐sectional friction profiles, in which how the friction varies with the number of scans can be clearly observed. d) Average friction force after tapping as well as the friction enhancement, averaged over the tapping area, exhibit a linear decrease with the number of scan images. e) The friction results obtained during the subsequent eight tapping processes demonstrate the stability and reliability of the proposed method for writing and erasing frictional information. Here $f_{B}$ and $f_{T}$ represent the friction force before and after tapping, respectively. $f_{B}-f_{T}$ therefore corresponds to the friction enhancement.

Data erasability is a critical aspect of information storage systems. In order to address this, we propose an in‐plane scanning method using the same AFM tip, analogous to erasing a blackboard, to effectively erase previously written frictional information. This erasing process involves conducting successive scans over the desired area, gradually diminishing the frictional patterns. To demonstrate the erasability capability, we performed friction force mapping during the first and seventh scans using a normal force of 22 nN and a scan velocity of 12 μm s^−1^, as depicted in **Figure** **3**a,b. In the first scan, the pre‐written square pattern is clearly visible, but progressively weakens with subsequent scans until it completely disappears by the seventh scan. The cross‐sectional friction profiles (Figure 3c), highlighted in Figure 3a,b, further exemplify the variations in friction as a function of the number of scans. Figure 3d then presents the calculated friction force averaged over the tapping ($F_{T}$) and intact area ($F_{B}$) and the change in friction ($F_{T}-F_{B}$) as a function of the number of scans. It is observed that the friction force without tapping remains constant throughout the scans, while the friction force with tapping exhibits a linear decrease with increasing scan number. This demonstrates the effectiveness of the tapping method in facilitating efficient data erasure. Furthermore, to enhance the efficiency of data erasure, increasing the normal load during the in‐plane scan can be implemented (refer to Figure S4, Supporting Information for further details).

We additionally investigated the rewritability of the proposed tapping methods, which is another crucial aspect for its potential application in information storage. To assess this, we initially patterned a square with dimensions of 500 nm and a tapping force of 17.5 nN. Subsequently, we conducted repeated scans across the square with a larger size of 3 μm, using a normal force of 3.5 nN and a scan velocity of 6 μm s^−1^, for seven times until the square pattern completely erased. Additionally, we repeated the write and erase process on the same region for a total of eight cycles. As shown in Figure 3e, the friction signal obtained in the first scan after tapping exhibited excellent stability. There were no significant changes in the friction signal observed during the subsequent eight tapping events, indicating the high stability and repeatability of the proposed out‐of‐plane tapping method (refer to Figure S2, Supporting Information for further details).

**Figure 4:**
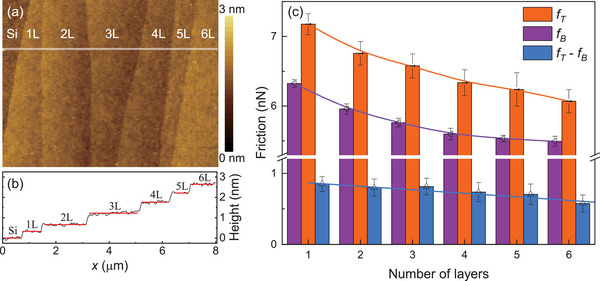
Thickness dependence of patterning. a) Topographic image of the thin graphene flake (forward scan). The regions marked Si, 1L, 2L, etc., correspond to the SiO_2_/Si substrate and where the flake is mono‐layer, bi‐layer, etc.; b) Cross‐sectional height profile on the flake; c) The measured friction and friction enhancement as a function of the sample thickness before and after the out‐of‐plan mechanical tapping.

The impact of sample thickness on the patterning process was also investigated. For this purpose, we intentionally selected a flake with different layers and obtained topographic AFM images, as shown in **Figure** **4**a. The regions marked Si, 1L, 2L, and so on, correspond to the SiO_2_/Si substrate and the areas where the flake is mono‐layer, bi‐layer, and so on. The cross‐sectional profile of the flake is presented in Figure 4b. In agreement with prior research,^[^ ^15^ ^]^ the intrinsic friction on the graphene surface without tapping decreases with increasing sample thickness. Similarly, the friction force after out‐of‐plane mechanical tapping also decreases with increasing sample thickness. Furthermore, the friction difference before and after tapping ($F_{T}-T_{B}$) also decreases with sample thickness, indicating that graphene patterning occurs only in thin flakes. We also performed a similar patterning process on a thick HOPG sample and observed no friction differences after tapping (for more details, please refer to Figure S3, Supporting Information). These results demonstrate the potential of the proposed tapping method for patterning thin graphene flakes with varying thicknesses.

**Figure 5:**
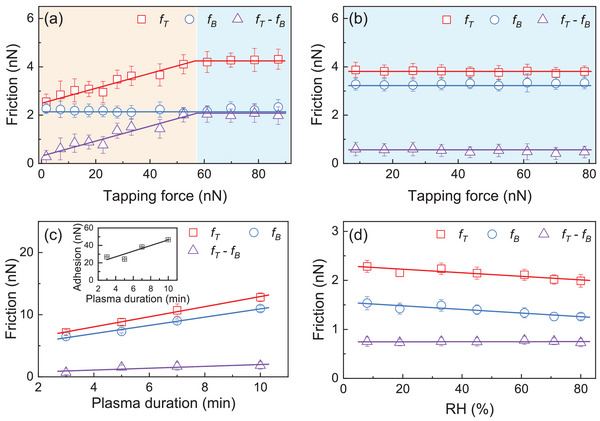
Tapping force, plasma pre‐treatment and environmental effects on friction. Friction force measured with the SiO_2_/Si substrate treated by the O_2_ plasma for a) 5 s and b) 30 s. c) The friction forces and friction enhancement increase with the duration of O_2_ plasma treatment on the tip. d) Both friction forces before and after tapping decrease with RH while the friction enhancement remains nearly invariant to RH.

Finally, we investigated the influence of tapping force, plasma pre‐treatment duration, and environmental conditions on patterning. **Figure** **5**a presents the measured friction force of graphene on a SiO_2_/Si substrate pre‐treated with O_2_ plasma for 5 s as a function of the tapping force $F_{p}$. Initially, increasing the tapping force $F_{p}$ results in a linear increase in the friction force $F_{T}$. However, as $F_{p}$ continues to increase, $F_{T}$ reaches a point where it becomes nearly independent of $F_{p}$. This behavior can be attributed to a stronger interaction force between the tip and the graphene sheet, allowing for greater modulation of friction. However, there is a limitation to the friction modulation if the tapping force is further increased. On the other hand, when the plasma pre‐treatment duration is extended to 30 s, $F_{T}$ remains nearly constant within a certain range of $F_{p}$. This indicates that when the graphene film is tightly bonded to the substrate, it becomes difficult to modulate the friction behavior through tapping alone.

The friction enhancement observed in our study is a direct result of the modulation of the graphene/substrate interface, which is influenced by the interaction between the tip and the graphene surface. To enhance the tip‐graphene interaction, we employed a pre‐treatment technique using oxygen plasma on the Si_3_N_4_ tip. This pre‐treatment serves two purposes: First, it effectively removes adsorbates from the tip surface, ensuring a clean contact with the graphene. Second, it introduces oxygen chemical groups onto the tip surface, promoting a stronger interaction with the graphene. We quantitatively characterized the increased interaction force between the tip and graphene using adhesion force measurements (insert of Figure 5c), which confirmed the enhanced tip‐graphene interaction after plasma pre‐treatment. As a result, we observed that both the friction force before and after tapping, as well as the friction enhancement, increased with longer plasma pre‐treatment duration (Figure 5c).

The present of water molecules can modulate the interaction between the tip and the graphene, we have conducted additional measurements under various conditions by altering the relative humidity of the environment from 8% to 80%. These measurements were performed using a Si_3_N_4_ tip with a tapping force of 7.5 nN. The results of these experiments demonstrated that both the friction forces before and after tapping exhibited a decreasing trend with increasing relative humidity. It is worth noting that previous studies have shown that RH does not strongly influence friction on the basal plane of graphene and highly oriented pyrolytic graphite (HOPG).^[^ ^31^, ^32^ ^]^ While we currently lack a clear understanding of the underlying mechanism, we speculate that the decrease in friction with increasing RH may be related to the presence of water molecules in the environment, which can influence the interaction between the tip and the graphene surface. Interestingly, we observed that the friction enhancement, which represents the difference between the friction forces before and after tapping, remained nearly unchanged across different relative humidity levels. This suggests that the modulation of friction achieved through our tapping method is robust and independent of environmental humidity conditions. Based on these observations, it is noteworthy to mention that enhancing the tip‐graphene interaction force through oxygen plasma pre‐treatment, increasing the number of tapping times, and using alternating tapping points can all contribute to improving the quality of the frictional patterns. These strategies provide means to optimize the friction modulation process and achieve desired patterning results. On the other hand, the tapping speed does not have a significant influence on the patterning process.

**Figure 6:**
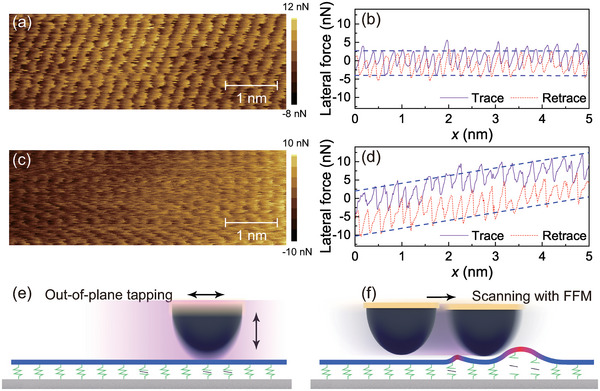
Atomically resolved friction measurements on graphene surfaces and schematic illustration of the friction enhancement mechanism. a,b) The representative atomic stick‐slip friction force maps and curves recorded before tapping; c,d)the similar results after. Image size, 5 nm × 5 nm; normal load, 0.16 nN; scan velocity, 40 nm s^−1^. For measurements before tapping, only the regular atomic‐scale stick‐slip has been observed. In contrast, for the measurements after tapping, the apparent lateral force increase can be observed. e) After the out‐of‐plane mechanical tapping, the binding strength between graphene and the substrate is weakened. f) During FFM scanning, the graphene that has undergone tapping exhibits increased deformability compared to the untapped graphene. This increased deformability is a result of the weakened binding between the graphene and the substrate due to the tapping process, which leads to a larger contact area and higher friction force.

In the following parts, we will discuss the possible mechanisms. To further investigate the underlying mechanisms, we have conducted atomic resolution stick‐slip measurements before and after tapping, as shown in **Figure** **6** (image size: 5 nm × 5 nm; normal load: 0.16 nN; scan velocity: 40 nm s^−1^). The top panels of Figure 6 display the recorded friction results before tapping, while the bottom panels show the friction results after tapping. Atomic‐level stick‐slip on graphene surface yields friction images with threefold symmetric patterns corresponding to the known symmetry of the lattice. As shown by the representative friction loops in Figure 6a,b, the tip exhibits clear, periodic stick‐slip motion, similar to that observed previously for graphene.^[^ ^15^ ^]^ For the measurements before tapping, only regular atomic‐scale stick‐slip has been observed. In contrast, for the measurements after tapping, the apparent lateral force strengthening (the lateral force at which each slip occurs slightly increases in magnitude during forward and backward scan, resulting in a tilted friction loop) can be observed, providing strong evidence of contact quality evolution.^[^ ^15^, ^22^ ^]^

Based on these observations, we propose a phenomenological model to explain the underlying mechanism. After the tapping process, the binding strength between the graphene and the substrate weakens due to the large adhesion force between the graphene and the Si_3_N_4_, as the binding energy between Si_3_N_4_/graphene is significantly greater than that between SiO_2_/graphene.^[^ ^33^, ^34^ ^]^ During FFM scanning, the graphene that has undergone tapping exhibits increased deformability compared to the untapped graphene. As a consequence, the contact area between the AFM tip and the tapped graphene is larger than that of the untapped graphene, leading to an increase in the friction force. To confirm this hypothesis, we performed tapping experiments on graphene using a SiO_2_ tip (see Figure S6, Supporting Information for further details). As expected, no noticeable change in friction was observed. The degenerated graphene/substrate interface undergoes slight recovery during the in‐plane scanning process.

## 4 Conclusion

In conclusion, we have introduced a novel approach to reversibly pattern graphene's frictional properties using out‐of‐plane mechanical tapping. The tapping process increases the friction force between the tip and the graphene film, which can be detected by FFM. This behavior is attributed to the change in interfacial binding strength between the graphene and substrate. The reversible frictional properties of graphene hold significant promise for its application as an information storage medium. With its high storage capacity and the ability to write and erase information directly, akin to a blackboard, graphene exhibits great potential for digital information storage with long‐term stability. Overall, our study opens up new avenues for controlling and tuning friction in graphene, and underscores the importance of understanding nanoscale friction for various technological applications.

## Conflict of Interest

The authors declare no conflict of interest.

## Author Contributions

W.W. conceived the study. W.W. and Y.Z designed the experiment(s). Y.Z. and W.W. conducted the experiment(s). Y.Z. and W.W. analyzed the results. All authors discussed the results and contributed to writing the manuscript.

## Supporting information

Supporting Information

## Data Availability

The data that support the findings of this study are available from the corresponding author upon reasonable request.
