# Supplementary material for: Controllable Friction on Graphene via Adjustable Interfacial Contact Quality
Source: Adv Sci (Weinh). 2023 Sep 3;10(30):2303013. doi: 10.1002/advs.202303013 (PMC10602576; doi:10.1002/advs.202303013)
Supplement: Supplementary file 1 — Supporting Information [file ADVS-10-2303013-s001.pdf]

ADVANCED SCIENCE
Open Access

Supporting Information

for *Adv. Sci.*, DOI 10.1002/advs.202303013

Controllable Friction on Graphene via Adjustable Interfacial Contact Quality

Wen Wang, Yu Zhang, Zhihong Li and Linmao Qian**

Controllable Friction on Graphene via Adjustable Interfacial Contact Quality (Supplementary Information)

Wen Wang[1*], Yu Zhang[1], Zhihong Li[2], Linmao Qian[1*]

[1] *School of Mechanical Engineering, Southwest Jiaotong University, 610031 Chengdu, China*

[2] *Key Laboratory of Microelectronic Devices and Circuits (MOE), Institute of Microelectronics, Peking University, 100871 Beijing, China*

1. Effects of tapping times on frictional patterning

The influence of tapping times was investigated in this study to understand how repeated tapping affects friction. A 500 nm×500 nm region was tapped with a predefined tapping force of 87.3 nN, allowing for modulation of friction. Figure S1 demonstrates this process, utilizing a tapping array of 20×20 points. Initially, as shown in Figure S1(a), a high friction region was introduced; however, the boundary of this region was not well-defined, and the extent of friction enhancement was limited. Subsequent tapping improved these issues, as depicted in Figure S1(b). To optimize the pattern and minimize the need for multiple tapping cycles, an alternating increase in tapping points was employed in our experiments.

2. Relaxation time effect on frictional patterning

The friction change induced by the mechanical tapping process has proven to be highly stable. While we did not conduct a systematic investigation into the relaxation effects, we have observed that patterns prepared more than one month ago remain clearly visible in the friction channel. This is exemplified by the black rectangles as shown in Figure S2. We believe that in the absence of external applied fields, these patterns can be maintained for an extended period.

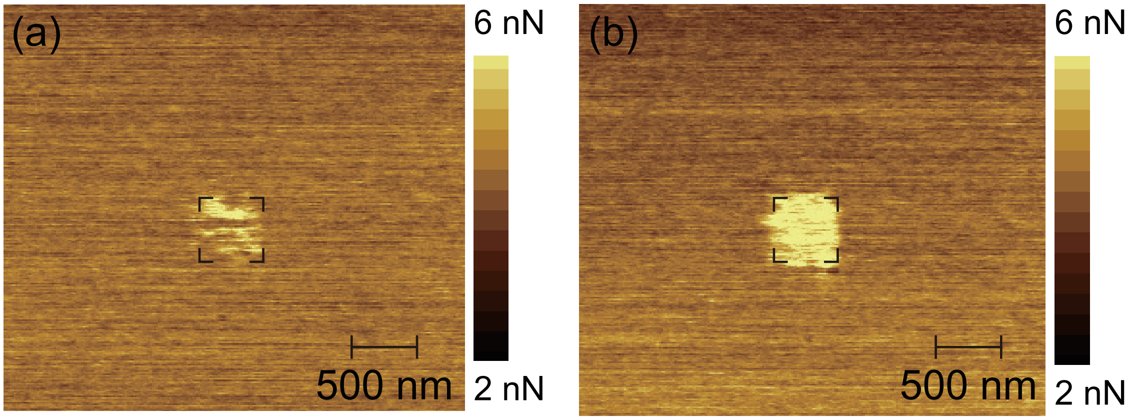

Figure S1: Effects of tapping times on frictional patterning. Friction image after (a) One-time tapping. (b) Two-times tapping. Tapping array is 20×20 points and pre-tapping force is 87.3 nN. Tapping are is 500 nm×500 nm

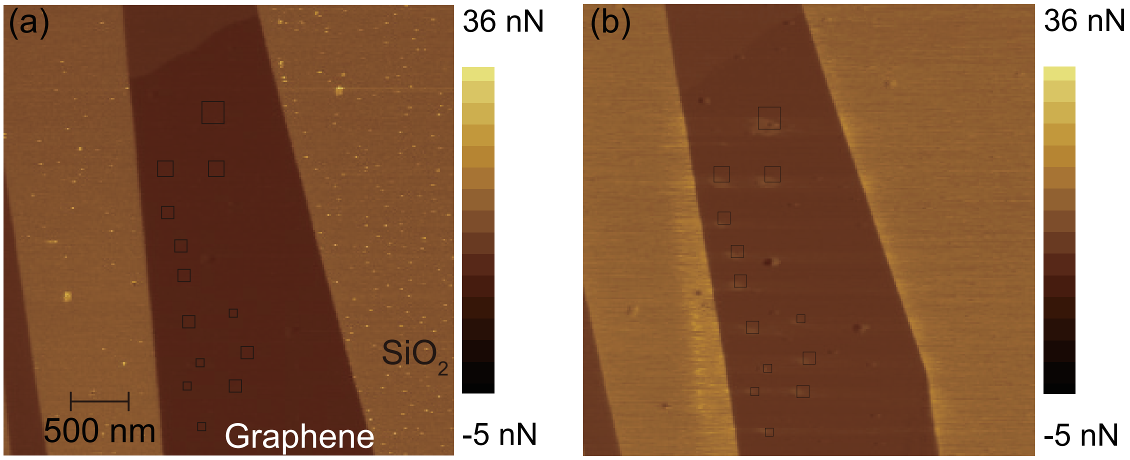

Figure S2: Relaxation time effect. (a) Large area frictional image of mono-layer graphene on SiO_2/Si substrate before patterning. (b) Frictional image of the patterned graphene after one month where the high friction pattern can be clearly observed as indicated by black rectangles.

3. In-plane scan

To validate the importance of out-of-plane mechanical tapping, we performed a similar patterning process by replacing the out-of-plane tapping with an in-plane scan. Firstly, we continuously scanned a small area on a graphene plane using the same Si_3N_4 tip with a normal force of 70 nN and a scan velocity of 2 μm/s. We then scanned a larger area to check for changes in friction. Fig. S3a shows the friction map of graphene on a SiO_2/Si substrate with a scan size of $5\mu m \times 5\mu m$. The white rectangles indicate the small in-plane scan area. Fig. S3b displays the friction map after the in-plane scan. No obvious changes in friction were observed, indicating that the change in friction is mainly caused by out-of-plane tapping.

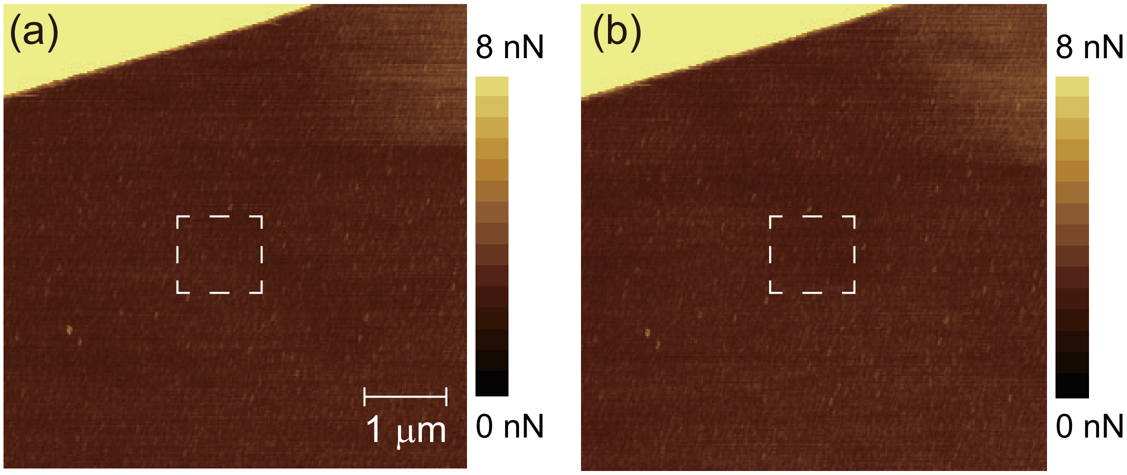

Figure S3: In-plane scanning on a graphene sample has no effect on the writing. (a) Friction force map before in-plane scanning. (b) Friction force after in-plane scanning.

4. Erase with different normal forces

To investigate the effect of normal force on the erasing (scanning) process, we carried out erasing measurements with different normal forces. As shown in Fig. S4a, it took five scans for the pattern to completely disappear at a small normal load, while only two scans were needed for a larger normal force (Fig. S4b). We conclude that increasing the normal force during scanning can speed up the erasing process.

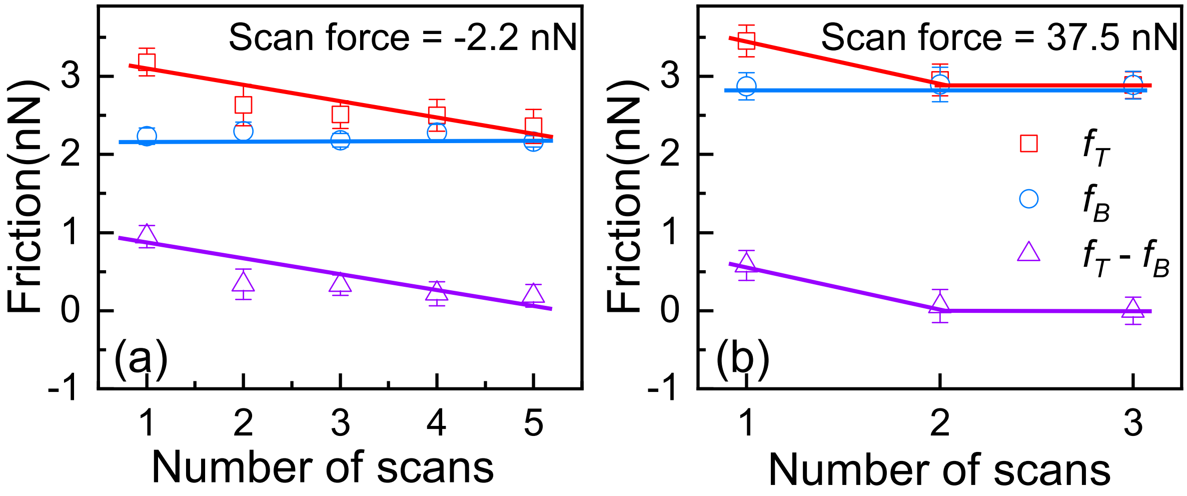

Figure S4: Erasing the pre-written pattern with reciprocal scanning using a normal force of (a) -2.2 nN and (b) 37.5 nN.

5. Tapping on a thick HOPG

We performed a similar patterning process on a thick HOPG sample using the same Si_3N_4 tip. To obtain a clean sample surface, the HOPG sample was freshly cleaved immediately before tapping. Fig. S5a and b show the topography and friction maps of the HOPG sample with a scan size of 2 μm $\times$ 2 μm and normal force of 1.1 nN. No obvious patterns were observed, except for some step edges introduced during the cleavage process. We then tapped on a small area of 650 nm $\times$ 650 nm with a tapping force of 98.5 nN, as indicated by the white rectangles. Fig. S5c and d show the topography and friction maps of the same region after tapping. No obvious changes in the topography and friction maps were observed. Therefore, the out-of-plane tapping process can only be used for thin graphene sheets.

6. Tapping with different tips

After exfoliation, graphene sheets stay on the SiO_2/Si substrate, and the interfacial contact quality between the graphene and the substrate degenerates due to the large jump-off force. To validate this assumption, we used a Si tip (PPP-LFMR from Nanosensors) to tap on the graphene.

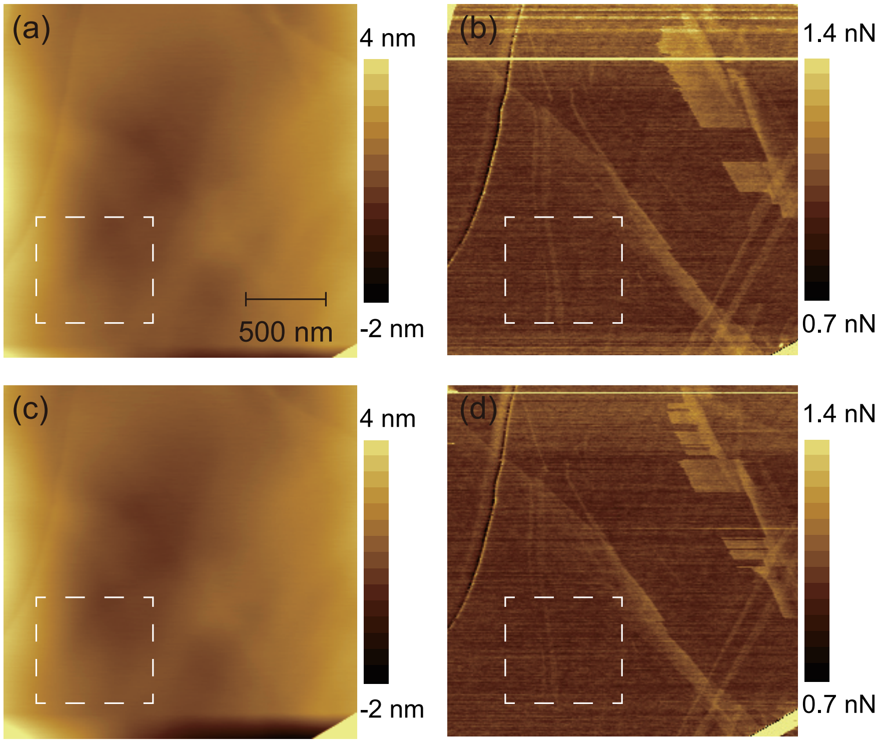

Figure S5: Topography (a) and friction (b) maps on a HOPG sample before tapping. Topography (c) and friction (d) maps on the same region after tapping. Scan size: $2\mu m \times 2\mu m$, normal force: 1.1 nN; Tapping area: 650 nm$\times$650 nm, tapping force: 98.5 nN.

It is important to note that the silicon tip oxidizes into a silicon oxide tip in the atmosphere, so we indeed used a silicon oxide tip for the experiment. Both the silicon oxide and Si_3N_4 tips had a similar radius of 10 nm. As expected, no obvious friction change was observed when using the silicon oxide tip (Fig. S6a and b). However, a friction difference was observed when using the Si_3N_4 tip (Fig. S6). Since the binding energy for Si_3N_4/Graphene is much larger than that for SiO_2/Graphene [1,2], the interfacial contact quality can be tuned during the retracting process. Therefore, we conclude that using a tip with a larger binding energy is a key factor in writing on graphene.

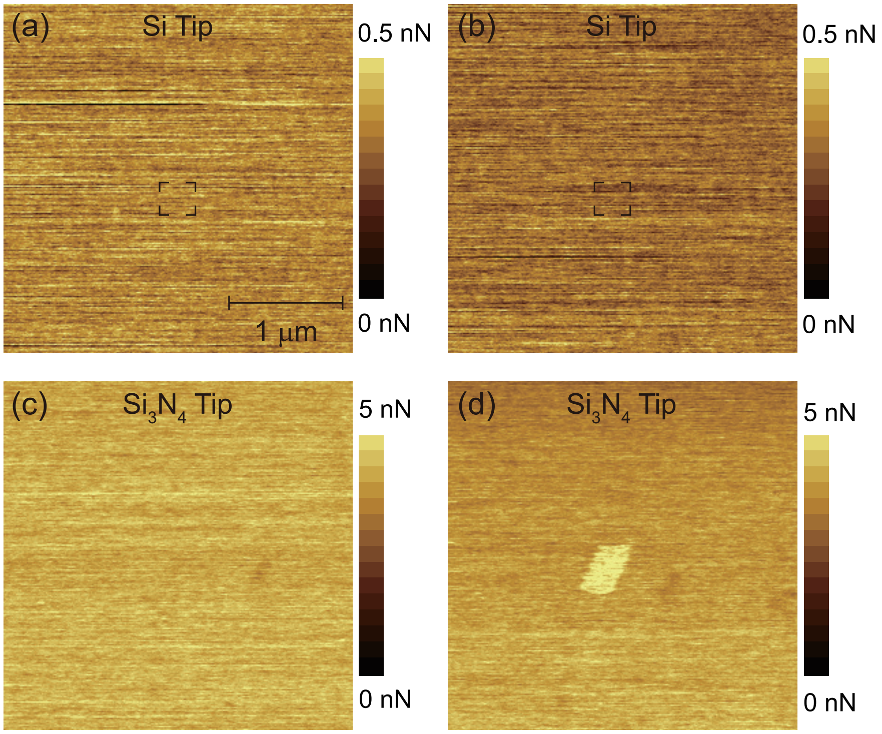

Figure S6: Friction maps before (a) and after (b) tapping by using a silicon oxide tip. Friction maps before (c) and after (d) tapping by using a Si_3N_4 tip.

7. Comparison with different storage media

In the study, we were able to achieve a minimum storage unit size of approximately 10 nm×10 nm using a tip with a radius of approximately 10 nm. The spacing between these storage units is

also 10 nm×10 nm. Consequently, each storage unit (one bit) occupies an area of 20 nm×20 nm. Based on these dimensions, the storage density per square inch is estimated to be approximately $(6.4516\ cm^2 / 400\ nm^2)/109 \approx 1613\ Gb/in^2$.

Currently, the primary storage media are Hard Disk Drives (HDD) [3], Heat-Assisted Magnetic Recording (HAMR) [4], 3D Flash [5], and Tape [6]. HDDs have a storage density that depends on several factors, including the disk material, magnetic head technology, track density, and number and size of magnetic heads. The commonly used storage density is typically less than 1000 GB/in^2. HAMR uses high anisotropy particles as storage media and can achieve a storage density of 2000 GB/in^2. 3D Flash storage density is often accompanied by upgrades in process technology and chip complexity, and high-density flash chips can achieve a storage density as high as 3838.7 GB/in^2. Tape storage technology has achieved a storage density of 317 GB/in^2 by using a 29-nm-wide tunneling magnetoresistance reader to study the recording performance of a new prototype magnetic tape based on vertically oriented strontium ferrite particles.

In addition, some new storage media are worth noting. Researchers used a nanopatterning technique called tip-enhanced near-field infrared nanolithography to pattern silk fibroin films for data storage [7]. Full-width grayscale and bilevel nano-patterns were fabricated with a minimum feature size of 35 nm and a pitch of 100 nm. As an optical storage medium, the silk drive can store digital information with a capacity of 64 GB/in^2. Silk protein can be easily functionalized by doping with various functional molecules and exhibits long-term stability under various harsh conditions. If silk storage devices can be mass-produced, the production cost of storage devices will be greatly reduced. Another promising development direction is DNA storage [8], which has an extremely high storage density. According to current technology, each gram of DNA molecule can store about 10^{18} bytes of data, equivalent to storing about 10^6 GB of data per square inch. This is hundreds of times higher than the storage density of the current most advanced hard disk and flash technologies. However, DNA storage has some disadvantages, such as slow synthesis speed, high synthesis cost, and poor real-time data reading.

By comparing the storage density of common storage media, such as HDDs and flash, and by calculating and analyzing the storage density of new storage materials, such as tapped graphene, silk proteins, and DNA, the results are shown in Figure S7.

Although the proposed mechanical tapping method can reach a high storage density, it is worthy to note that the wring and reading speed is still slow so far. Typically, the time required to record one vertical force curve depends on the piezo response, and in our measurements, it typically takes approximately 0.25 seconds using the AFM/FFM system (MFP-3D, Oxford). For example, if we write a bit with 10 nm$\times$10 nm by using the proposed methods in this manuscript with one-time tapping, we need 0.25 s, which corresponds to the 4 bit/s. Similarly, for reading the maximum scan speed can set to 100 μm/s, which means the read of a bit needs 0.1 ms corresponding to the reading speed of 100 bit/s. Both values are much smaller than HDDs and SSDs.It is important to note that these values were obtained using a commercial AFM system, and the reading and writing speed can be further improved by developing a custom system specifically designed for high-speed operations.

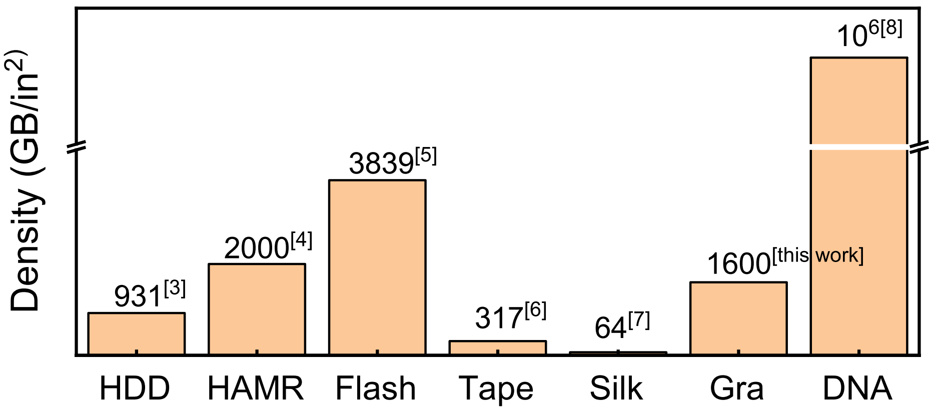

Figure S7: Comparison of storage densities of different storage media.

Supplementary References:

1. Cui, Z. et al. Research on the electronic properties of graphene/β-si3n4 (0001) heterojunction. Vacuum **184**, 109904 (2021).

2. Nguyen, T. C., Otani, M. & Okada, S. Semiconducting electronic property of graphene adsorbed on (0001) surfaces of sio 2. Physical review letters **106**, 106801 (2011).

3. Gao, K. Architecture for hard disk drives. IEEE Magnetics Letters **9**, 1–5 (2018).

4. Hono, K. et al. Heat-assisted magnetic recording media materials. MRS Bulletin **43**, 93–99 (2018).

5. Inaba, S. 3d flash memory for data-intensive applications. In 2018 IEEE International Memory Workshop (IMW), 1–4 (IEEE, 2018).

6. Furrer, S. et al. 317 gb/in 2 recording areal density on strontium ferrite tape. IEEE Transactions on Magnetics **57**, 1–11 (2021).

7. Lee, W. et al. A rewritable optical storage medium of silk proteins using near-field nano-optics. Nature Nanotechnology **15**, 941–947 (2020).

8. Zhirnov, V., Zadegan, R. M., Sandhu, G. S., Church, G. M. & Hughes, W. L. Nucleic acid memory. Nature materials **15**, 366–370 (2016).
